# RNASeq Analysis of *Aedes albopictus* Mosquito Midguts after Chikungunya Virus Infection

**DOI:** 10.3390/v11060513

**Published:** 2019-06-04

**Authors:** Ravi kiran Vedururu, Matthew J. Neave, Mary Tachedjian, Melissa J. Klein, Paul R. Gorry, Jean-Bernard Duchemin, Prasad N. Paradkar

**Affiliations:** 1 CSIRO Health & Biosecurity, Australian Animal Health Laboratory, Geelong 3220, Australia; Ravikiran.Vedururu@csiro.au (R.k.V.); Matthew.Neave@csiro.au (M.J.N.); Mary.Tachedjian@csiro.au (M.T.); Melissa.Klein@csiro.au (M.J.K.); Jean-bernard.Duchemin@csiro.au (J.-B.D.); 2 School of Applied Sciences, RMIT University, Bundoora 3083, Australia; 3 School of Health and Biomedical Science, RMIT University, Bundoora 3083, Australia; Paul.Gorry@rmit.edu.au

**Keywords:** Chikungunya, *Aedes albopictus*, RNASeq, Host–pathogen interactions

## Abstract

Chikungunya virus (CHIKV) is an emerging pathogen around the world and causes significant morbidity in patients. A single amino acid mutation in the envelope protein of CHIKV has led to a shift in vector preference towards *Aedes* *albopictus*. While mosquitoes are known to mount an antiviral immune response post-infection, molecular interactions during the course of infection at the tissue level remain largely uncharacterised. We performed whole transcriptome analysis on dissected midguts of *Aedes albopictus* infected with CHIKV to identify differentially expressed genes. For this, RNA was extracted at two days post-infection (2-dpi) from pooled midguts. We initially identified 25 differentially expressed genes (*p*-value < 0.05) when mapped to a reference transcriptome. Further, multiple differentially expressed genes were identified from a custom *de novo* transcriptome, which was assembled using the reads that did not align with the reference genome. Thirteen of the identified transcripts, possibly involved in immunity, were validated by qRT-PCR. Homologues of seven of these genes were also found to be significantly upregulated in *Aedes aegypti* midguts 2 dpi, indicating a conserved mechanism at play. These results will help us to characterise the molecular interaction between *Aedes albopictus* and CHIKV and can be utilised to reduce the impact of this viral infection.

## 1. Introduction

Arboviruses, such as the dengue, chikungunya, and Zika viruses, are a significant burden on public health systems worldwide. These viruses are transmitted by mosquitoes and can cause high morbidity and mortality, with dengue alone causing more than 300 million infections per year [1]. First identified and described in 1955 in a report on an outbreak at the border of Tanzania and Mozambique in 1952, chikungunya virus (CHIKV) is an enveloped, positive sense RNA virus belonging to the alphavirus genus in the *Togaviridae* family [2,3]. CHIKV infection in humans causes high febrile illness, cutaneous exanthema and debilitating and often prolonged arthralgia [4,5,6].

While *Aedes aegypti* is the traditional vector for CHIKV, *Aedes albopictus* has been observed to be implicated in viral transmission ever since the Reunion Island outbreak in 2005–2006 [7]. A single amino acid change in codon 226 of the E1 gene, which encodes for the envelope protein of the virus, has improved the fitness of the Indian ocean lineage CHIKV in *Aedes albopictus* [8]. As an invasive species, *Aedes albopictus* has been expanding its traditional habitat of tropical and sub-tropical regions to much cooler temperate regions. *Aedes albopictus* also survives in favourable microhabitats, even in winter and freezing temperatures [9]. These factors have further increased the risk of CHIKV to cause outbreaks in areas where mosquito-borne viral diseases are uncommon, such as Northern America and temperate Europe [10,11].

For a mosquito to become infective, the virus needs to cross two critical barrier tissues: the midgut and salivary glands. These infection barriers can be influenced by multiple factors, including viral factors, such as viral glycoproteins, or vector factors, such as the presence of a viral receptor, host replication factors and the microbiome composition of the midgut [12]. When an adult female *Aedes albopictus* mosquito is exposed to CHIKV in the process of blood feeding, the virus infects the midgut usually in a matter of hours [13,14,15]. Inside mosquitoes, after feeding, the blood meal moves down to the midgut where the virus must contact epithelial cells before digestion of the blood meal and formation of the peritrophic matrix takes place. Following the successful infection of the midgut, the virus must then overcome midgut escape barriers to disseminate to other tissues, such as the haemocoel [16]. From the haemocoel, the virus makes its way to the salivary glands of the mosquito. Once the virus is detected in the saliva, the mosquito is considered to be infective and a competent vector [17,18].

Since vector competence of the mosquito is initially dependent on infection of the midgut (Virus crossing the first critical barrier), understanding the molecular interactions between virus and mosquito midgut becomes essential. Although previous studies have used transcriptome-based approaches to identify mosquito–virus interactions, tissue-specific responses during the course of infection are not well-understood [19,20,21,22,23,24]. Here, using next-generation sequencing, we characterised the whole transcriptome response at the midgut in *Aedes albopictus* in response to CHIKV infection.

## 2. Materials and Methods

### 2.1. Chikungunya Virus

Chikungunya virus isolate 06113879 (Mauritius strain), isolated from a viraemic traveller who returned to Australia in 2006, was obtained from the Victorian Infectious Diseases Reference Laboratory (VIDRL), Melbourne [25]. The isolate was passaged in Vero cells (ATCC, Manassas, VA, USA) four times, followed by once in C6/36 (*Aedes albopictus* larval cell line) followed again by Vero cells, and was then used for the experiments. A TCID50 assay was performed on Vero cells to determine viral titer.

### 2.2. Aedes Mosquito Rearing, Infection and RNA Extraction

All experiments were performed under biosafety level 3 (BSL-3) conditions in the insectary at the Australian Animal Health Laboratory, CSIRO. Insectary conditions were maintained at 27.5 °C and 70% in relative humidity with a 12 h light and dark cycle. Female mosquitoes (5–8 days old) were challenged with a chicken blood meal spiked with CHIKV (1 in 100 dilution of stock virus, TCID_50_ 1.5 × 10^9^/mL) through chicken skin membrane feeding. After one hour, the mosquitoes were anesthetised with CO_2_, and the blood-fed females sorted and kept in 200 mL cardboard cup containers at 27.5 °C, 70% humidity and a 14:10 day:night photoperiod for 2 days with 10% sugar solution ad libitum. For controls, females were fed with blood mixed with media supernatant from an uninfected Vero cell culture. Midguts were dissected at 2 dpi and were stored in 50 µL of Qiagen RLTplus buffer with 5–10 silica beads (1 mm) at −80 °C.

Bead beating was performed on MP Biomedicals FastPrep -24™ homogeniser, 3 cycles, speed: 6.5 m/s, 45 s each cycle. RNA was extracted using the RNeasy^TM^ kit (Qiagen, Chadstone, Victoria, Australia), and cDNA was generated by using random hexamers and Superscript-III reverse transcriptase (Thermo Fisher Scientific Inc. Scoresby, Victoria, Australia) following the manufacturer’s protocols.

Complementary DNA (cDNA) generated from the RNA extracted from the midguts pools was tested for CHIKV viral RNA using an in-house-designed qRT-PCR, using primers specific for the E1 gene (Table S1). For RNASeq data validation, adult *Aedes albopictus* female mosquitoes were infected with CHIKV as described above. RNA was extracted from the midguts of 5 infected mosquitoes 2 dpi, and cDNA was generated individually by previously described protocols. cDNA from the midguts of 5 uninfected mosquitoes was used as controls. Mosquitoes from multiple generations but approximately of the same age were used for the experiments.

For comparison of differential gene expression in *Aedes aegypti*, RNA was collected from the midguts of 6 mosquitoes and infected with CHIKV through blood feeding at 2 dpi. RNA that was extracted from the midguts of 5 uninfected mosquitoes was used as a control. cDNA was generated as described above and qPCR was performed with *Aedes aegypti* gene-specific primers.

### 2.3. qPCR

Quantitative PCR (qPCR) was performed using gene-specific primers and 18s rRNA specific primers as internal controls were used to validate the expression changes of 8 targets. Midgut tissue from three infected and control mosquitoes was used for validation of 5 long non-coding RNAs (lncRNAs) by the above-mentioned method.

qPCR was performed on an Applied Biosystems QuantStudio™ 6 using the SYBR Green Master Mix: SYBR Premix Ex Taq II (Tli RNase H Plus) (Takara- Scientifix Pty Ltd., Clayton, Victoria, Australia). The following cycling conditions were used with a melt curve at the end, 30 s at 95 °C, 40 cycles of 5 s at 95 °C and 30 s at 60 °C. The baseline and Ct values were calculated automatically using the supplied QuantStudio™ Software (Thermo Fisher Scientific Inc. Scoresby, Victoria, Australia), and the ∆∆Ct values were calculated using the average ∆Ct value of controls and 18s rRNA as a reference.

### 2.4. RNASeq and Viral Genome Sequencing

To identify the genes involved in the initial infection stage, midguts from CHIKV-infected *Aedes albopictus* mosquitoes at 2 dpi from 6 mosquitoes were pooled together for RNA extraction to obtain sufficient material. This was also required to avoid using a low-RNA input RNAseq kit, which would likely introduce bias during the PCR amplification stage. The pool size was kept as low as possible to retain information on biological variations.

Libraries for RNASeq were prepared using Nugen’s Ovation Universal RNASeq kit, following manufacturer’s specification with a minor modification in the HL-dsDNAse treatment. During first strand synthesis with DNase treatment, HL-dsDNase from Thermo Fisher Scientific was used in our library preparation, along with the 10x buffer supplied that their protocol used. The libraries were pooled and sequenced on a single lane of Hiseq-2500 (Macrogen Inc., Seoul, South Korea) to generate 2 × 100 bp reads. The fastq files were deposited in NCBI’s (National Center for Biotechnology Information) Sequence Read Archive (SRA Accession ID: SRP140387).

The Qiagen QIAseq FX Single Cell RNA Library kit was used for Illumina library preparation from total RNA extracted from CHIKV-infected Vero cell culture supernatant using the RNeasy^TM^ kit (Qiagen, Chadstone, Victoria, Australia). The library was sequenced on a Miniseq (Illumina, Scoresby, Victoria, Australia), with the mid-output kit (300 cycles) generating 2 × 150 bp paired-end reads. The resultant fastq files were quality-trimmed and assembled to a consensus sequence on the CLC Genomics workbench v9.5.2. The sequence was annotated and submitted to GenBank (MH229986).

### 2.5. Differential Gene Expression and Gene Ontology Analysis

Quality trimming of the raw sequences was performed using Trimmomatic v0.36. The reads were aligned to the CHIKV reference sequence (GenBank ID: MH229986) to assess the infection status using Hisat2 v2.0.5 [26].

Following removal of *Gallus gallus* (GenBank assembly accession: GCA_000002315.3) reads (due to chicken blood feeding) using SAMtools v1.3.1, the remaining reads were aligned to the *Aedes albopictus* Foshan strain genome sequence (AaloF1) from Vectorbase using Hisat2, and the resultant SAM file was sorted and converted into a BAM file using SAMtools [27,28].

On Galaxy virtual lab v1.4.6.p5, featureCounts v1.4.6-p5 was used to quantify aligned transcripts from the sorted BAM files with default parameters for paired-end reads, and DESeq2 v2.11.38 was used to obtain differentially expressed genes between the controls and infected samples by using default parameters [29,30].

Using Trinity v2.3.2, a custom de novo transcriptome was built by combining the unaligned reads from midguts (D2) [31,32]. This transcriptome was used as a reference genome. edgeR was used to align the reads, measure the transcript counts and quantify the differentially expressed genes. The differentially expressed genes were annotated using BlastX [33,34].

The gene ontology (GO) IDs of the differentially expressed genes were obtained using the Biomart tool, and topGO analysis was performed to identify the Molecular Functions (MF), Biological Processes (BP) and Cellular Components (CC) that were either enriched or depleted in the differentially expressed genes [35,36]. Using topGO’s classic algorithm, and based on *p*-values generated using Fisher’s exact method, differentially expressed genes were grouped based on their ontologies. Enrichment percentage was calculated as the ratio of the number of times particular genes in the pathway were differentially expressed compared to the expected number by chance.

## 3. Results

### 3.1. Whole Genome Sequencing of CHIKV

The chikungunya virus isolate 06113879 was used to infect *Aedes albopictus* mosquitoes. Although a 559 bp portion of the E1 gene has been published (GenBank ID: EU404186.1), full genome sequencing of this isolate has not been performed, which can inform us about the genotype of this virus as well as be used as a reference for removing the virus reads that are detected during the RNASeq analysis.

Whole genome sequencing performed on the CHIKV isolate, after single Vero passaging, using the Illumina MiniSeq, resulted in about 8.5 million quality-trimmed, paired-end reads. The assembly resulted in an 11,929 bp long consensus sequence (MH229986), which perfectly matched the previously published 559 bp portion of the E1 gene from this isolate.

The viral consensus sequence was most similar (Identity: 11,705/11,985 (97.7%), Similarity: 11,705/11,985 (97.7%), Gaps: 245/11,985 (2.0%)) to the CHIKV strain LR2006_OPY1 (GenBank: KT449801.1) and had the E1-A226V mutation, indicating that this isolate also belongs to the same lineage as the CHIKV that caused the La Reunion outbreak in the Indian ocean in 2006.

### 3.2. RNASeq

To determine differentially expressed genes, five pools of midguts (six mosquitoes per pool) collected at 2 dpi from infected mosquitoes were created. For controls, three pools of midguts were used. Initially, to determine the infection status of these tissues, qRT-PCR was performed using CHIKV-specific primers. Based on these results and qualitative and quantitative requirements of the Nugen Ovation universal RNAseq kit, two control and three infected midgut pools were used to prepare sequencing libraries.

The sequencing of five libraries on a single lane of HiSeq-2500 resulted in 37 million to 170 million reads each. After quality trimming, reads mapping to the chicken genome were discarded to remove those originating from undigested chicken blood. The remaining reads were aligned to the *Aedes albopictus* reference genome with an average alignment of 62.25%. The read alignment to the CHIKV reference sequence (MH229986) also confirmed that all the three infected libraries contained viral reads, while the two control libraries did not, as shown in Table 1.

### 3.3. Differential Expression and TopGO Analysis

**Figure 1:**
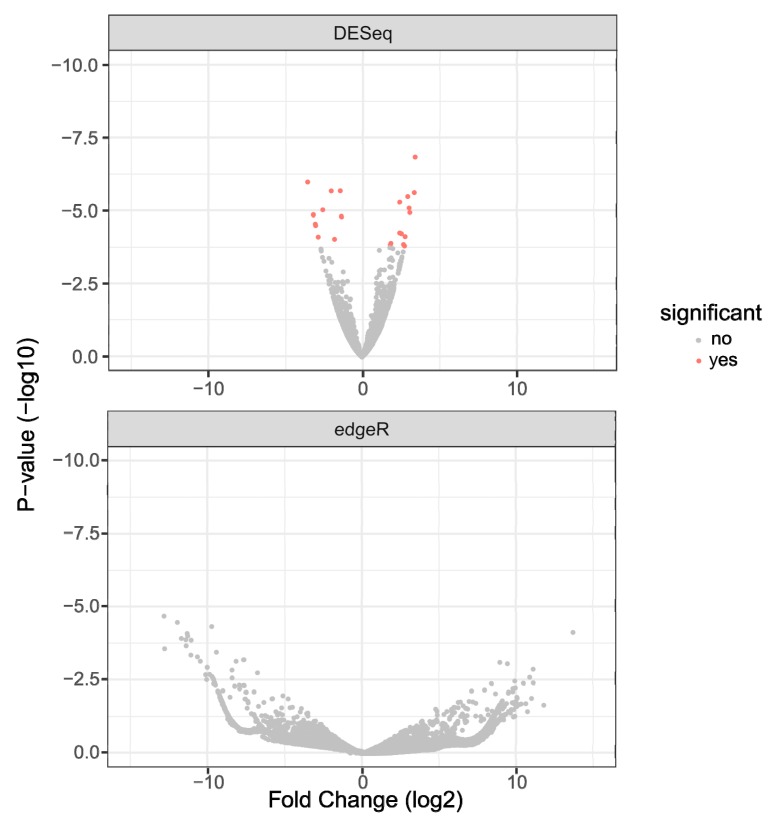
Volcano plots from DESeq2 and edgeR for 2 dpi differential gene expression; Volcano plots from DESeq2 (top panel) and edgeR (bottom panel) of differentially expressed genes from 2 dpi. DESeq2 was performed by aligning reads to the *Aedes albopictus* reference genome. edgeR analysis was done on reads that did not align to the reference genome and were aligned to the custom transcriptome.

Differentially expressed genes were identified using DESeq2 (for reads aligned to the RefSeq genome) and edgeR (for the de novo transcriptome built with aligned reads) and were plotted as Volcano plots (Figure 1). The results showed 25 genes to be differentially expressed as detected by DESeq2 (Up: 14 and Down: 11). edgeR did not show statistically significant (False Discovery Rate < 0.05) differential expression of genes due to a sub-optimal number of control libraries. However, targets from this dataset were chosen based on raw *p*-values for validation. The complete list of genes and transcripts that were differentially expressed with *p*-values of less than 0.05 is provided in Supplementary Information S2. The fasta files that were obtained as output for the custom transcriptome assembly are provided in Supplementary Information S3.

**Figure 2:**
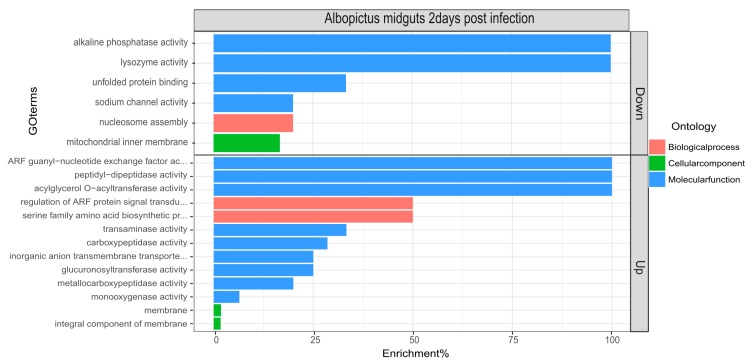
topGo enrichment comparison in differentially expressed genes. Enrichment analysis of down- and upregulated genes in the midguts of *Aedes albopictus* mosquitoes in response to Chikungunya virus (CHIKV) at 2 dpi. Enrichment % is calculated as the ratio of ‘significant’ (Number of times the gene ontology number is observed as differentially expressed) to ‘expected’ (Number of times the gene ontology number is expected based on observation in control samples) gene numbers.

To determine the biological processes and molecular functions of the differentially expressed genes, gene set enrichment analysis and ontology was performed using topGO (Figure 2). As expected, several biological and molecular processes were significantly affected during CHIKV infection.

The differentially regulated biological processes were metabolic (*p*-value = 0.012) and the serine family amino acid biosynthetic process (*p*-value = 0.026). The differentially regulated molecular functions, likely of interest from an innate immune response point of view, were lysozyme activity (downregulated, *p*-value = 0.028), alkaline phosphatase (downregulated, *p*-value = 0.033) and carboxypeptidase activity (upregulated, *p*-value = 0.042).

Apart from protein-coding genes, five long non-coding RNA (lnc RNA) were also detected to be differentially expressed. These were >200 bp long sequences with no apparent reading frames and, when searched using Blast against the published *Aedes albopictus* genomes on NCBI, showed high similarity to sequences annotated as long non-coding RNAs.

### 3.4. RNASeq Data Validation on qRT-PCR

Based on previously known immune involvement, we selected eight genes (one aligned to the reference genome and seven from the custom transcriptome) and five long non-coding RNA (from the custom transcriptome) for validation by qRT-PCR (Table 2). For this, midguts were dissected from five individual CHIKV-infected adult *Aedes albopictus* female mosquitoes (2 dpi). Midguts from uninfected mosquitoes were used as controls.

As the results showed, for *Aedes albopictus* mosquitoes, among the eight protein coding transcripts chosen for validation, the expression pattern for six targets was concordant, and the expression pattern for two targets was discordant, between RNASeq and qRT-PCR. Among the five tested long non-coding RNAs, two were concordant. All seven identified homologue targets, tested in *Aedes aegypti* midguts at 2 dpi, were upregulated as compared to uninfected controls. In *Aedes albopictus*, only five of these targets were upregulated as per qRT-PCR.

## 4. Discussion

Chikungunya virus is a re-emerging alphavirus causing a high morbidity with long-term arthralgia in infected patients. Previous studies have taken approaches to understand the interaction between chikungunya virus and the *Aedes aegypti* vector, although these focused only on certain known genes and pathways [37]. However, considering the switch in vector preference towards *Aedes albopictus* by the Indian Ocean strains and invasive nature of this mosquito species, it is paramount to characterise the interaction between CHIKV and the new vector.

Previous studies with whole transcriptome analysis in mosquito vectors have used either whole mosquitoes or a cell culture [19,21,22,23,38]. Our objective here was to study the vector–virus interaction specifically at the midgut, which is the first barrier site to understand the factors that play a critical role in determining mosquito vector competence.

In the current study, unbiased transcriptional analysis was performed on midgut tissues collected from lab-reared adult female *Aedes albopictus* mosquitoes post CHIKV infection. The gene expression patterns, when compared to uninfected samples, revealed the transcriptional changes that are likely to be in response to the viral infection. Our analysis revealed that at 2 dpi in the midgut, most of the transcriptional changes were related to metabolism. An analysis of molecular functions revealed that while lysozyme activity and alkaline phosphatase were downregulated, carboxypeptidase activity was upregulated. Indeed, lysozymal and carboxypeptidase pathways have previously been implicated in innate immune responses [39,40,41,42,43].

One of the genes that was identified to be upregulated, and later validated by qRT-PCR, was a homolog of the Neimann Pick 2 (*NPC2*) gene, which in humans encodes for an intracellular cholesterol transporter. Loss of function mutations in the *NPC2* gene lead to a lysosomal disorder known as Niemann–Pick disease type C, which causes an increased accumulation of lipids in cellular compartments and leads to cell death [44,45]. In many viral infections, replication and assembly of the viral particles occur at the lysosomal and other intracellular membrane-bound organelles, including DENV infection in its vector *Aedes aegypti* [46]. NPC2 has previously been shown to be involved in the replication and transport of several viruses, such as ebolavirus, vesicular stomatitis virus and influenza A virus, into and out of cells [47,48,49]. Interestingly, we detected a significant upregulation of the NPC2 homolog both in *Aedes albopictus* as well as *Aedes aegypti* midguts 2 days post infection with CHIKV. Recent studies have shown that Imipramine-based inhibition of the NPC2 protein results in severely diminished CHIKV replication in human fibroblast skin cells [50]. The fact that the NPC2 gene is significant and essential for CHIKV replication in humans and is significantly upregulated in the mosquito midgut after infection implies that an evolutionarily conserved mechanism may be at play and presents possible therapeutic opportunities in clinical treatment.

Multiple prior publications have also shown that the RNAi pathway is one of the major pathways involved in antiviral responses in insects [16,37,51,52]. We did not detect differential expression of the traditional innate-immune response pathways, such as JAK/STAT, IMD and Toll, consistent with previous studies with CHIKV infection of *Aedes aegypti* [37]. It is possible that the regulation of this pathway either does not occur at the transcriptional level or the proteins involved are ubiquitously expressed and not differentially regulated. It is also possible that the time point we selected did not coincide with RNAi activation.

Long non-coding RNAs are RNA molecules that are over 200 bp long and do not contain an open reading frame. They are produced by RNA Polymerase II and are processed like mRNAs by the cellular mechanism, including polyadenylation. lncRNAs have been implicated with involvement in multiple viral infections, including dengue and Influenza [39,53,54]. The mechanism of their involvement is complex, with pro or anti-viral activity [55,56,57,58,59,60]. Our understanding of lncRNA involvement in host viral interactions is still limited. Our results showed an increase in the number of lncRNAs in *Aedes albopictus* midguts after CHIKV challenge, suggesting a role during the infection. The functional significance of this differential regulation remains to be seen.

While the DESeq2 analysis identified statistically significant differential gene expression, edgeR, performed on the custom transcriptome that was assembled using reads that did not align to the published *Aedes albopictus* reference genome, was not able to find significantly altered genes with the set FDR < 0.05. This may be due to the small sample size.

To address this issue, we performed RT-qPCR-based validation of transcripts that appeared to be differentially expressed based on raw *p*-values, on a fresh set of individual mosquito midguts (five controls vs. five infected). RT-qPCR showed significant differential expression among all the transcripts tested, including in the targets from the edgeR dataset. These genes also showed differential expression when tested in CHIKV-infected *Aedes aegypti*, further validating their significance during infection.

The targets from the edgeR dataset, chosen for qPCR-based validation, were selected based on evidence from previously published data regarding their involvement in innate immune pathways. Seven out of eight protein coding targets and all of the five lncRNAs validated on RT-qPCR were from the edgeR dataset, and most showed varying levels of differential expression. Functional characterisation of the identified genes may help us to decipher the results and understand their role in mosquito–virus interactions.

Our results showed differences in gene expression pattern between *Aedes aegypti* and *Aedes albopictus*. While all of the seven tested genes in *Aedes aegypti* were upregulated, only five were upregulated in *Aedes albopictus* with the remaining two being downregulated. This may indicate differences in host–pathogen interactions between the two species of mosquitoes when infected with CHIKV.

Overall, our results showed significant changes in the transcriptome of *Aedes albopictus* mosquitoes after CHIKV infection, with the identified genes being involved in multiple cellular processes. This study examined differential gene expression at the midgut (the first critical barrier site) in infected *Aedes albopictus* mosquitoes. This study can be utilised in determining potential pro-viral and antiviral host factors and, in turn, will be helpful in reducing the high impact of CHIKV infections by targeting the vector *Aedes albopictus*.

## Figures and Tables

**Table 1:** RNASeq next-generation sequencing (NGS) data summary. The total number of obtained reads, the reads’ alignment percentage to the reference genome and the percentage of reads aligned to the chikungunya virus genome.

	Total Reads (millions)	% Mapped to RefSeq Genome	% of CHIKV Reads
Infected MG 1	170.65	60.83%	0.01%
Infected MG 2	37.13	60.46%	0.02%
Infected MG 3	38.02	65.28%	0.10%
Control MG1	44.77	61.53%	0.00%
Control MG2	44.72	63.15%	0.00%

**Table 2:** List of genes validated by qRT-PCR and comparison of expression fold changes.

Genes	*Aedes albopictus*		*Aedes aegypti*
	LogFC (RNAseq)	Expression Fold Change (qRT-PCR)	Expression Fold Change (qRT-PCR)
*ARF GTPase-activating protein GIT2*	−8.7	0.76(↓)	39.74(↑)
*NPC2 homolog*	6.29	5.35(↑)	11.63(↑)
*Mucin-22/FLO-11-like*	−8.61	0.16(↓)	7.34(↑)
*Translocon-associated protein subunit delta*	8.82	1.88(↑)	40.18(↑)
*ATP-dependent RNA helicase dbp2*	−9.07	6.53(↑)	26.22(↑)
*Uncharacterized gene coding for Sina and RING_Ubox domains containing protein*	8.84	1.51(↑)	41.07(↑)
*E3 ubiquitin-protein ligase MARCH6*	−9.04	1.03(↑)	28.65(↑)
*Ankyrin repeat domain-containing protein 44*	−5.56	0.5(↓)	No homologue/Not tested
*PREDICTED: Aedes albopictus uncharacterized LOC109424229 (LOC109424229), ncRNA*	−11.22	2.14(↑)	No homologue/Not tested
*PREDICTED: Aedes albopictus uncharacterized LOC109622934 (LOC109622934), ncRNA*	−7.13	0.06(↓)	No homologue/Not tested
*PREDICTED: Aedes albopictus uncharacterized LOC109423409 (LOC109423409), ncRNA*	−9.07	0.73(↓)	No homologue/Not tested
*PREDICTED: Aedes albopictus uncharacterized LOC109414360 (LOC109414360), ncRNA*	−9.53	1.02(↑)	No homologue/Not tested
*PREDICTED: Aedes albopictus uncharacterized LOC109424229 (LOC109424229), ncRNA*	−8.98	0.04(↓)	No homologue/Not tested

## Supplementary Materials

The following are available online at https://www.mdpi.com/1999-4915/11/6/513/s1, Table S1: List of primers, S2: List of differentially expressed genes, S3: Custom transcriptome output.
