# Supplementary material for: RNASeq Analysis of Aedes albopictus Mosquito Midguts after Chikungunya Virus Infection
Source: Viruses. 2019 Jun 4;11(6):513. doi: 10.3390/v11060513 (PMC6631752; doi:10.3390/v11060513)
Supplement: Supplementary file 1 [file viruses-11-00513-s001.zip › S1 Supplementary information.docx]

Supplementary information for:

RNASeq analysis of *Aedes albopictus* mosquito midguts after chikungunya virus infection

# Table A: List of Primers

| Primer | Sequence |
| --- | --- |
| CHIKV_E1_FOR | AAGAGCGATGAACTGCGCCGTAG |
| CHIKV_E1_REV | CTGGTACCTCGCATGACATGTC |
| AALF004300_D8_FOR | CACGAACCGAACGGATATG |
| AALF004300_D8_REV | TTCTGCTGCTGCTGTTG |
| AALF008354_D8_FOR | CGTGTCCTTCTACCAAATCC |
| AALF008354_D8_REV | TCTTGCGCTTCAGCTTAC |
| AALF011899_D8_FOR | GCTCGATGCGAGATAAGAAG |
| AALF011899_D8_REV | CTCGGTATCTTCAACGGTAATC |
| AALF012324_D8_FOR | GGCACCATCAATAGGGTAAC |
| AALF012324_D8_REV | CCTTCGAGCCAAACTCTATG |
| AALF012634_D8_FOR | CGGGAGATTTACGAGGTTTC |
| AALF012634_D8_REV | GCGTTCTTCCCTTCATCTC |
| AALF016505_D8_FOR | GGAATGTGGCAATGTGAATAC |
| AALF016505_D8_REV | GATCACTCGATCGGCATAAG |
| AALF016704_D8_FOR | ACTGACGTTCCCTTCAAAC |
| AALF016704_D8_REV | GCTCGATCGACTTCATCTTC |
| AALF021910_D8_FOR | GTGAACCTCATTCCGGATAC |
| AALF021910_D8_REV | CTTTCACCTCGGTCCAATC |
| AALF023547_D8_FOR | GAACCATCGTGGAAGAAGAG |
| AALF023547_D8_REV | CATCAGCTTCAGAGCCATATC |
| AALF025245_D8_FOR | CGGAGAAGCAGTTGGTATTC |
| AALF025245_D8_REV | CGATCGAGTGGAAGTCTTTG |
| AALF026574_D8_FOR | GAGGTGCTCGTTACATCTTG |
| AALF026574_D8_REV | GCAATGGGTGGTACCTTATC |
| DN129476_D8_FOR | GTGCTCTTCCGATCTTTCTC |
| DN129476_D8_REV | GAAGGTATCGTCGAGTTCAAG |
| DN131737_D8_FOR | GGGTCTTGAGCGAATGTATC |
| DN131737_D8_REV | TGCCGCATACTTGTAGTTATC |
| DN131885_D8_FOR | ACTCTGGTGTACCCTTATCC |
| DN131885_D8_REV | TTCCTAGTGGTAGTGATCGAG |
| AALF020406_D2_FOR | GTACGATAGGACAGCCAAAC |
| AALF020406_D2_REV | GTCTCCTTTCTCCCGATTTG |
| DN102975_D2_FOR | GAATTGTGCGTACGATTTGG |
| DN102975_D2_REV | GGTGCATGGTGTATGAAATTG |
| DN109582_D2_FOR | GGAGAAGAGCTATCCACCTATC |
| DN109582_D2_REV | TTCCCTTGCACACTGAAAC |
| DN109663_D2_FOR | TCCGTTACTTCGGTTGATTG |
| DN109663_D2_REV | ATGCCACAGATCCTGAAAC |
| DN110186_D2_FOR | CATGGATCAACTCGGAGATTC |
| DN110186_D2_REV | ATCTCCTCTTCCCGCTTAC |
| DN110327_D2_FOR | ACACACGCCTCGCTGATATG |
| DN110327_D2_REV | AAGATCAATAGGGAAATTTCAAAGC |
| DN110556_D2_FOR | CATCGCCTACTACGTGATTC |
| DN110556_D2_REV | GCAAATGACCGTCCAAATG |
| DN46100_D2_FOR | CACGCGAATACGTTAAATTCC |
| DN46100_D2_REV | ATTTCGCAGCACAATTTCAG |
| 18srRNA_Fwd | CGGCTACCACATCCAAGGAA |
| 18srRNA_Rev | GCTGGAATTACCGCGGCT |
| Aegypti_D2_Mucin_qPCR_FOR | CTCAAACGGAGACCTCAAGC |
| Aegypti_D2_Mucin_qPCR_REV | TGGGTCGTCTCGGTAGAATC |
| Aegypti_D2_NPCV2_qPCR_FOR | TAGTTCCGGTCAAGGAATGC |
| Aegypti_D2_NPCV2_qPCR_REV | TCGCTGACACTGAAGTCCAC |
| Aegypti_D2_DN110556_qPCR_FOR | CTGAAATGTCCAGGATGTGC |
| Aegypti_D2_DN110556_qPCR_REV | GCATTTCGGTCAGCTTCTTC |
| Aegypti_D2_DN110186_qPCR_FOR | ACGGAATTCACCCTGAAGTG |
| Aegypti_D2_DN110186_qPCR_REV | CCAGCTGACCTGGTACTTGC |
| Aegypti_D2_DN102975_qPCR_FOR | TAGCGTTTGGGAGCACCTAC |
| Aegypti_D2_DN102975_qPCR_REV | CGACAGATTCACGTGTTTGG |
| Aegypti_D2_DN46100_qPCR_FOR | TGAACGATCCGGACTTTAGC |
| Aegypti_D2_DN46100_qPCR_REV | GCCCACAGCATTAGGAGAAC |
| Aegypti_D2_DN110327_qPCR_FOR | ACAGGCACCTGGTGGTAAAG |
| Aegypti_D2_DN110327_qPCR_REV | CGAATGGCTTCTTCTGGAAC |
